# Optimization of Man Power Deployment for Covid-19 Screening in a Tertiary Care Hospital: A Study of Utility of Queuing Analysis

**DOI:** 10.1017/dmp.2021.228

**Published:** 2021-07-21

**Authors:** Shakti Kumar Yadav, Garima Singh, Namrata Sarin, Sompal Singh, Ruchika Gupta

**Affiliations:** 1 Department of Pathology, Hindu Rao Hospital, Delhi, India; 2 Division of Cytopathology, ICMR-National Institute of Cancer Prevention and Research, Noida, India

**Keywords:** queuing model, health system, patient loss, waiting time, saturation

## Abstract

**Objectives:**

The recent Covid-19 pandemic has burdened the healthcare facilities, especially in the presence of limited infrastructure. We aimed at applying a queuing model to the Covid-19 screening area so as to optimize the screening services and ensuring that no patient is refused the service.

**Methods:**

The mean arrival time of patients, number of physicians, mean screening time and queue characteristics were observed and entered in the M/M/c/K queuing model using R programming to optimize the number of physicians required in the screening area.

**Results:**

Considering the mean arrival of 7 patients in 10 minutes and screening of 3 patients in 10 minutes by 1 physician, 2 physicians were assigned. At this capacity, the probability of saturation of the system was 15% with patient loss rate of 1.05 per 10 minutes. Queuing simulation with 3 physicians reduced the patient loss rate to 0.013 per 10 minutes and a saturation probability of 0.2%. However, an increase of arrival rate from 10 to 20 led to an early saturation of the system.

**Conclusion:**

Queuing models offer an opportunity for the healthcare providers and hospital administrators to optimize patient care services, especially in critical areas with an ever-changing situation such as the current pandemic.

## Introduction

The recent outbreak of the 2019 novel Coronavirus or Severe Acute Respiratory Syndrome Coronavirus 2 that originated from Wuhan City in China has assumed global epidemic proportions. In such situations, timely delivery of services in the existing limited infrastructure is paramount to ensure service is still rendered to all those who require it.^1^ Although the mortality rate in this pandemic is low, the disease is highly infectious.^2^ Due to the possibility of asymptomatic carriers, public health measures including avoiding close contact with individuals having respiratory symptoms, frequent hand washing and social distancing have to be implemented.^3^ Since health services constitute an essential component of control measures against such infectious agents, these public health measures apply equally to the health care workers and individuals visiting the health centers. It also needs to be kept in mind that the infrastructure (space), facilities (testing, isolation, and treatment), and personnel (doctors, nurses, paramedics) in any given health facility are finite leading to queues and these cannot be increased overnight to combat such a sudden epidemic or pandemic. Hence, mathematical modeling techniques come in handy to assist the hospital management and policymakers in ensuring optimum and judicious use of the available infrastructure and manpower while complying with the disease containment measures. Queuing analysis is one such modelling technique that can be useful in an appropriate allocation of personnel (infrastructure being non-modifiable) to reduce the queue length and ensuring that no patient is refused service.^1^ Queuing models include certain assumptions for the arrival rate and service rate of a system and allows mathematical derivation of the number and type of servers required.^5^ A typical queuing system comprises of the population from where customers (patients) arrive, number of customers arriving per unit of time, queue length restriction if any, number of servers (physicians), and the queue discipline used. The queue discipline is the order in which the customers or patients in a queue are attended to. The discipline is usually first-in-first-out; though triage is also used in emergency rooms of hospitals.^1^ The queuing model to be applied in a given situation depends on the assumptions, finite or infinite source of patients and the capacity (finite or infinite) of the system. A queuing model written as the ‘M/M/c/K’ includes the first M for arrival distribution (for example, the Poisson distribution describing the number of events in an interval of time),^5^ the second M for service time (for example, the exponential distribution), c for number of servers and K for queue length restriction. Information about the population being finite or infinite and queuing discipline used may be appended, if required, to this nomenclature. The mathematical formulae used in queuing analysis are based on Little’s law, L = λW, where L denotes the average number of customers in the system, λ (lambda) stands for average arrival rate into the system and W is the average amount of time spent by a customer in the system.

Queuing models have been applied in a variety of fields such as service centers, airport terminals, manufacturing systems, and communication networks. In healthcare too, queuing analysis has been resorted to for understanding and mitigation of congestion at crucial areas such as emergency departments or intensive care units (ICU).^4^ Hospitals usually present as an ‘infinite source model’ where the number of customers or patients are independent and their arrival is assumed to follow a Poisson process. In a Poisson process model for a series of events, the average time between each event is known but the exact timing is random, hence, the arrival of an event is independent of the event prior to it.^1^ At the same time, most of the service points in hospitals work with a ‘finite capacity’ leading to ‘reneging,’ that is patients leaving the queue and hospital without being attended to and ‘balking’, where the patient registers displeasure of the waiting time and leaves the hospital. However, healthcare services, especially the ones at crucial points need to strive to keep the reneging and balking at a minimum, and ensure that majority and if possible all patients coming to their facility are attended to with minimal waiting. Hence, application of queuing theory and techniques can serve to improve the healthcare service delivery in the existing infrastructure by optimizing the manpower and facility utilization. In order to maintain the recommended distance between 2 patients in keeping with the WHO guidelines on physical distancing, the maximum capacity of the screening room in our hospital was fixed, hence in our queuing problem, the system has a limited capacity (K). As a result of this, the present study was aimed to identify the optimal number of physicians in the screening area, given the space constraint, to ensure that service is rendered to each patient entering into the system.

## Materials and Methods

This cross-sectional observational study was conducted in the area designated for screening and triage of COVID-19 patients at a tertiary care hospital in Delhi. All patients arriving at this facility were screened for the presence of symptoms indicative of the COVID-19 infection (structured questionnaire and screening for vitals such as temperature, pulse rate, and respiratory rate) in order to triage them for sample collection. To avoid exposure to the research personnel involved in the study, the observations were made from a separate room through CCTV camera and no intervention was made by the research team. The observations were taken for 10 consecutive days from 9 AM to 4 PM while 10 minutes was taken as the unit time for the queuing analysis.

**Figure 1.**
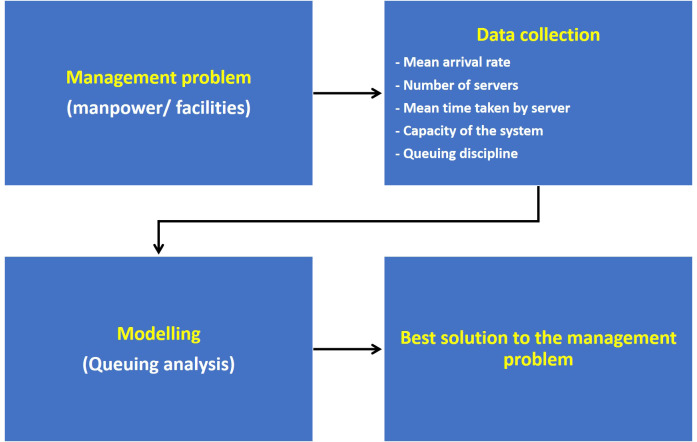
Flowchart depicting the algorithm used in the mathematical modelling in the present study.

The variables observed in this study include mean arrival rate of patients in the triage room, number of physicians attending patients (i.e., number of servers), and the mean screening time. The mean arrival rate per unit time (M) was determined by the number of patients registered at the registration counter. Screening time (M) was calculated from the time taken by a physician to interact with a patient and triage the patient. The c (number of physicians) is the dependent variable, which was varied in the model while K (queue length restriction) was fixed. These variables were then fed to the M/M/c/K model of queuing analysis and the resulting variables of queuing analysis such as server utilization rate, number of patients in the system and in the queue of triage area, and the probability of having the maximum allowed number of patients in the triage area were noted. This algorithm is depicted in Figure 1 and the coding is available on request.

Following the WHO guideline on social distancing, with respect to the recommended distance between 2 patients, the maximum capacity of the triage room (i.e., the system in terms of queuing analysis) in the hospital was 20 patients.

### Statistical Analysis

An open-source software, R programming was used for statistical modeling and calculation of the probabilities. Keeping the M, M and K constant, different scenarios with a variable number of servers (c) were considered for the calculation of queuing analysis probabilities. The patient loss rate, i.e. rate at which the patient leaves the system without being seen by the physician, mean patient waiting time before being screened by the doctor, and mean number of patients in the triage area were calculated for the various scenarios with different number of physicians. The optimal number of servers was determined to allow for minimum patient loss and average patient waiting time with appropriate number of patients in the system such that the probability of saturation is minimized.

Since this study was a mathematical modelling attempt with no intervention from the study researchers on the screening and triage being performed by the physicians, ethical clearance was not sought. The study was approved by the Institutional Review Board.

## Results

Our observations revealed that, on an average, a single physician was able to screen and triage 3 suspected COVID-19 patients in 10 minutes (unit time). The average arrival rate in our system was 7 patients in every 10 minutes. Hence it was intuitively decided to assign 2 physicians to screen and triage the patients for COVID-19.

We applied the MMcK queuing model in R programming to the observations and the following results were obtained from the software:

1. At the present arrival rate, the probability of having 20 patients in the screening and triage area was 15%.
2. The physician occupancy (server utilization rate) was 99.25% in any given unit of time (10 minutes).
3. The mean number of patients in the system was 14.91, out of which 12.9 were waiting in the queue while 2 were being examined by the physicians.
4. The average time spent by each patient in the system was 25 mins (2.5 x 10 mins), out of which an average of 21.7 mins (2.17x 10 mins) was spent in the queue by each patient.

The patient loss rate was calculated by multiplying the arrival rate by the probability of saturation of the system. In our system with 2 physicians, the patient loss rate was about 1 patient per unit time (7 x 0.15 = 1.05). This is to say that every 10 minutes, 1 patient was likely to leave the system without being evaluated by the physician.

Following the above observations, further simulation was performed using a variable number of servers (c), i.e., physicians in the screening and triage area. The results of the simulation are summarized as:

### 

#### Patient loss rate

**Figure 2.**
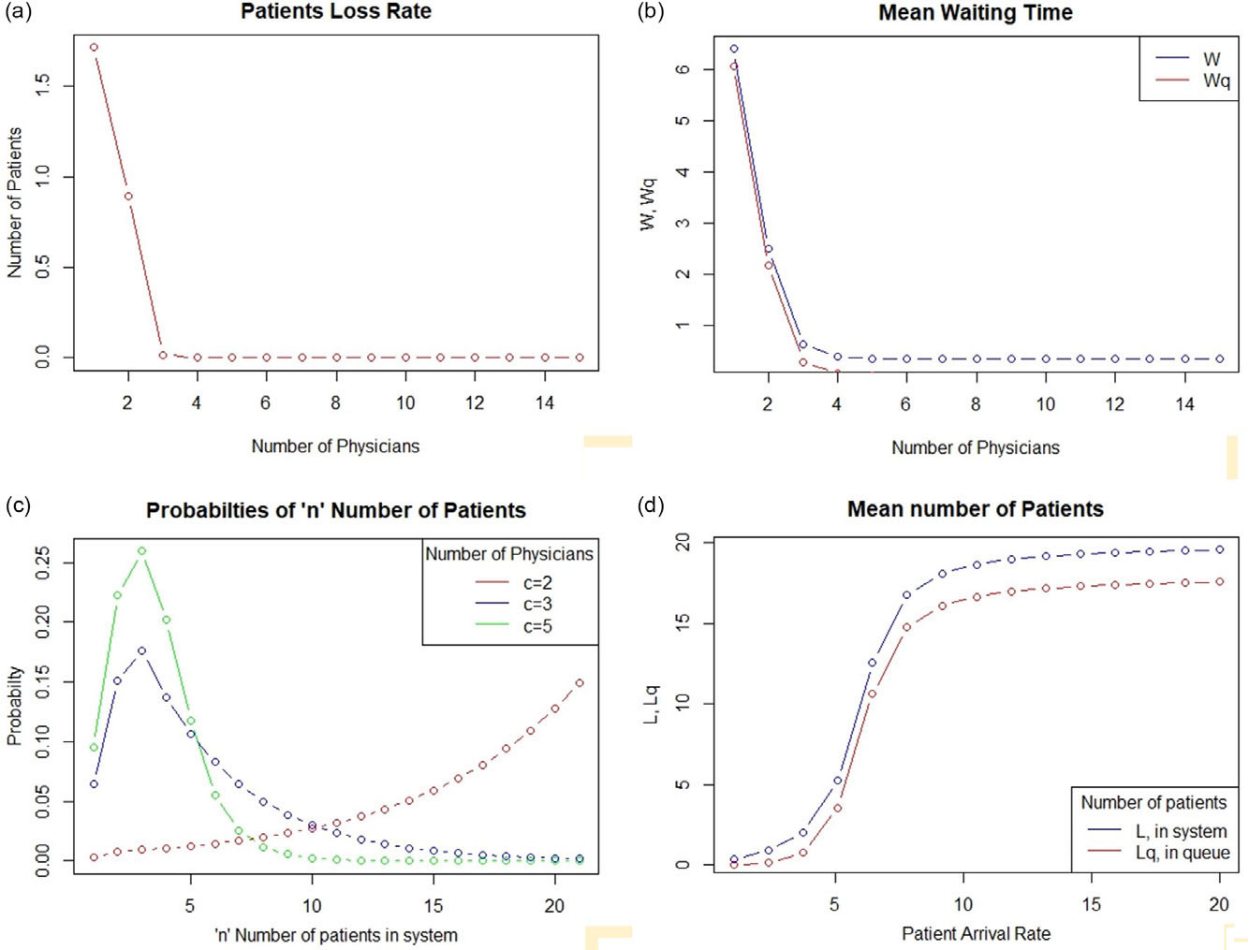
Graphical representation showing the effect of number of servers (physicians) on the patient loss rate (a), and the patient waiting times (b). Simulation for the effect of number of servers (c =2, 3, 5) on probability of “n” patients in system (c), and the effect of various arrival rates on the number of patients on in the system (d), is also depicted.

The rate of patient loss doubled if only 1 physician was available for screening the patients as compared to 2 physicians. However, the rate of patient loss could be nearly 0 (7 x 0.002 = 0.0134) if the number of physicians was increased to 3 or more (Figure 2a).

#### Mean waiting time for patients

If the number of physicians was reduced to 1, the mean waiting time would increase to more than 60 minutes. However, with 3 physicians, the mean waiting time could be significantly reduced to less than 10 mins (0.287 x 10 = 2.87), as depicted in Figure 2b.

#### Number of patients in the system

The probabilities of “n” number of patients in the system (waiting or being assessed) with various numbers (c = 2, 3, 5) of physicians are shown in Figure 2c. Increasing the number of physicians to 3 could reduce the probability of having a saturated system, i.e. having about 20 patients in the screening and triage area to 0.2% [p (n=20) is 0.002, i.e., 0.2%], thereby reducing the probability of refusal of service to patients.

#### Simulations using variable patient arrival rate

Since the situation of the novel coronavirus disease is fluctuating daily, the simulation was also performed using variable patient arrival rate. With 2 servers (physicians), the number of patients in the system could rapidly increase to full capacity even at an arrival rate of 10 patients every 10 minutes (Figure 2d).

#### Simulations using variable service rate

**Figure 3.**
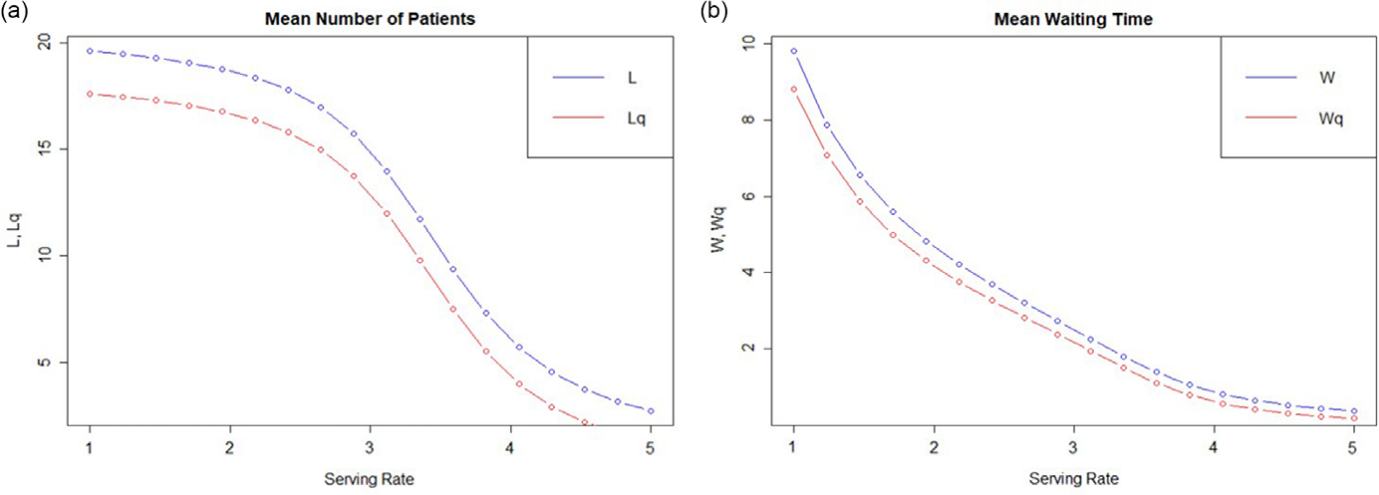
Graphical representation of simulation showing the effect of service rate on the number of patients on in the system (a), and patient waiting times (b).

We also simulated using variable patient service rate, that is, the number of patients screened in the unit time of 10 minutes, while having 2 physicians (servers) attending to them. The number of patients in the system and in the queue would fall drastically if the physicians were able to screen more patients in the same unit time (Figure 3a). The mean waiting time for the patients in the system and in the queue is also likely to reduce to approximately 0 with the patient service rate approaching 5 or more (Figure 3b).

## Discussion

In the present study, the observation area was a crucial spot in the wake of the ongoing pandemic and hence, this modelling was undertaken. Given the infinite source and finite capacity of the screening and triage area, the M/M/c/K queue model was used, for which it is assumed that all the arrivals at the time of queue being full are lost. A similar model is the M/M/1/K queue model where only 1 server or doctor attends to the arriving patients. However, in the presence of this infectious disease pandemic, patients could not be allowed to crowd in the screening area, the M/M/c/K model was used to determine the optimum number of physicians needed to ensure minimal reneging and balking, and at the same time, maintain social distancing between patients in waiting.

A study of the application of this model in emergency and ICU of a general hospital in Zimbabwe showed that in the presence of a limited waiting area in the emergency department, the addition of 1 doctor could reduce the number of patients in the queue from 3.87 to 0.3 along with a reduction in their average length of stay in the emergency ward. Similarly, the patients waiting in the queue reduced from 8.3 to 0.31 in the ICU.^1^ In the present study, a simulation was done using the M/M/c/K model, the patient loss rate could be reduced from 1.05 per 10 minutes (with 2 physicians in the screening area) to nearly 0 when physicians were increased to 3. The mean waiting time could also be brought down from 21.7 minutes to less than 10 minutes. Given the finite capacity system, the probability of the screening area getting saturated to its full capacity reduced from 15% (with 2 physicians) to 0.2% (with 3 physicians). Hence, our simulation demonstrated that the addition of just 1 physician in the screening and triage area could benefit the patients immensely.

The application of queuing models in infectious diseases has been limited. For instance, the arrival rate and service rate may be modified to signify the infection rate and recovery, or death rate, respectively to understand the transmission dynamics of infectious disease and help the authorities in planning control measures.^6^ The present study offers another avenue where queuing models and simple mathematical calculations can be done to optimize patient services. Simple observations of the arrival rate, waiting time and patient loss are required to apply the queuing theory. Simulation techniques provide the opportunity to hospital administrators to decide upon the number of servers or physicians that could minimize the waiting time and patient loss while maintaining efficiency and measures to reduce contact exposure to the patients as well as the physicians.

### Implications of the Present Study

The present study highlights that simple modelling techniques can help improve the medical practice and health service delivery especially at crucial points and during times of social emergencies like epidemic or pandemic. The results of the present study can be used by policymakers at the hospital administration level to optimize healthcare services in the presence of the available constraints of manpower and facilities. Further research in the field of mathematical modelling would be helpful in refining this technique.

## Conclusion

In conclusion, queuing models and their application to healthcare services can help in optimizing service delivery in varied situations, including infectious diseases. Since the epidemiology of a highly infectious disease such as the 2019 novel Coronavirus changes drastically within a short time, queuing theory can be effectively utilized to make quick decisions about the optimization of facilities and personnel for effective control measures.

## Data Availability

Data and code available on request.
